# Association of Antenatal COVID-19–Related Stress With Postpartum Maternal Mental Health and Negative Affectivity in Infants

**DOI:** 10.1001/jamanetworkopen.2023.2969

**Published:** 2023-03-14

**Authors:** Susanne Schweizer, Jack L. Andrews, Karina Grunewald, Levi Kumle

**Affiliations:** 1 Department of Psychology, University of Cambridge, Cambridge, United Kingdom; 2 School of Psychology, University of New South Wales, Kensington, New South Wales, Australia; 3 Department of Experimental Psychology, University of Oxford, Oxford, United Kingdom

## Abstract

**Importance:**

Antenatal stress is a significant risk factor for poor postpartum mental health. The association of pandemic-related stress with postpartum outcomes among mothers and infants is, however, less well understood.

**Objective:**

To examine the association of antenatal COVID-19–related stress with postpartum maternal mental health and infant outcomes.

**Design, Setting, and Participants:**

This cohort study was conducted among 318 participants in the COVID-19 Risks Across the Lifespan study, which took place in Australia, the UK, and the US. Eligible participants reported being pregnant at the first assessment wave between May 5 and September 30, 2020, and completed a follow-up assessment between October 28, 2021, and April 24, 2022.

**Main Outcomes and Measures:**

COVID-19–related stress was assessed with the Pandemic Anxiety Scale (score range, 0-4, with higher scores indicating greater COVID-19–related stress). The 8-item Patient Health Questionnaire (score range, 0-3, with higher scores indicating more frequent symptoms of depression) was used to measure maternal depression at each time point, and the 7-item General Anxiety Disorder scale (score range, 0-3, with higher scores indicating more frequent symptoms of anxiety) was used to measure generalized anxiety at each time point. At follow-up, postpartum distress was assessed with the 10-item Postpartum Distress Measure (score range, 0-3, with higher scores indicating greater postpartum distress), and infant outcomes (negative and positive affectivity and orienting behavior) were captured with the Infant Behavior Questionnaire (score range, 1-7, with higher scores indicating that the infant exhibited that affect/behavior more frequently).

**Results:**

The study included 318 women (mean [SD] age, 32.0 [4.6] years) from Australia (88 [28%]), the US (94 [30%]), and the UK (136 [43%]). Antenatal COVID-19–related stress was significantly associated with maternal postpartum distress (β = 0.40 [95% CI, 0.28-0.53]), depression (β = 0.32 [95% CI, 0.23-0.41]), and generalized anxiety (β = 0.35 [95% CI, 0.26-0.44]), as well as infant negative affectivity (β = 0.45 [95% CI, 0.14-0.76]). The findings remained consistent across a range of sensitivity analyses.

**Conclusions and Relevance:**

The findings of this cohort study suggest that targeting pandemic-related stressors in the antenatal period may improve maternal and infant outcomes. Pregnant individuals should be classified as a vulnerable group during pandemics and should be considered a public health priority, not only in terms of physical health but also mental health.

## Key Points

**Question:**

Is antenatal COVID-19–related stress associated with postpartum maternal mental health and infant outcomes?

**Findings:**

In this cohort study of 318 mothers in Australia, the UK, and the US, antenatal COVID-19–related stress was significantly associated with poor postpartum maternal mental health outcomes and increased negative affectivity among infants.

**Meaning:**

This study suggests that mitigating pandemic-related stressors during pregnancy should be a global public health priority.

## Teaser

This cohort study uses data from the COVID-19 Risks Across the Lifespan study to examine the association of antenatal COVID-19–related stress with postpartum maternal mental health and infant outcomes.

## Introduction

The Centers for Disease Control and Prevention classify pregnant individuals as a vulnerable group during pandemics.^1^ The classification is based on risks posed to physical maternal and infant health outcomes. However, emergent data from the current COVID-19 pandemic illustrates that the pandemic’s toll may be particularly high in terms of mental health. A recent meta-analysis found that during the COVID-19 pandemic, the worldwide prevalence of antenatal depression was 25.6% and the worldwide prevalence of antenatal anxiety was 30.6%.^2^ Compared with prepandemic norms, these rates are heightened, with pregnant individuals reporting significant increases in clinical levels of depression and anxiety during the COVID-19 pandemic.^3,4,5^

COVID-19–related stress is a likely factor associated with these increased rates of maternal antenatal mental health problems. Antenatal stress has been associated with poor postpartum maternal mental health^6^ and higher levels of behavioral and cognitive problems in infants.^7^ For example, one study found that greater stress among expectant mothers during the pandemic was associated with higher negative affect among infants aged 3 months.^8^ Maternal postpartum stress has also been associated with negative infant outcomes, including reduced positive affectivity and poorer orienting behavior, during the COVID-19 pandemic.^9^ Several other studies have also retrospectively assessed COVID-19–related stressors and found these stressors to be associated with postpartum maternal and infant outcomes. In particular, high levels of retrospectively reported, pandemic-related parental stress have been associated with higher postpartum maternal anxiety^10^ and infants’ surgency (ie, activity level and expression of pleasure) at 3 months.^11^

Although efforts have been made to examine the association of antenatal stress with maternal and infant outcomes during the pandemic, the existing literature is limited in several ways. First, most studies have been cross-sectional, precluding inferences of causal associations between antenatal stress and postpartum outcomes. Relatedly, when antenatal COVID-19–related stress was assessed, it was predominantly measured retrospectively, which increases the likelihood of reporting bias and is likely to be influenced by maternal postpartum experiences. Moreover, studies that captured COVID-19–related stress frequently used unvalidated measures without reporting psychometric properties. Although this is unsurprising given the urgency to gather data on COVID-19–related stressors during the pandemic, it does impose limitations on the conclusions that can be drawn from such findings. Finally, many of the studies were conducted in a single geographic location and included small sample sizes, significantly limiting their generalizability.

Drawing on data from the COVID-19 Risks Across the Lifespan (CORAL) study, a cohort study investigating the association of the COVID-19 pandemic with individuals’ mental health and cognition, we sought to comprehensively assess whether COVID-19–related stress experienced during the antenatal period was associated with postpartum maternal and infant outcomes. Our study was well placed to overcome the limitations highlighted because it was conducted across 3 countries (the UK, the US, and Australia), captured pregnancy status early in the pandemic, included a validated measure of COVID-19–related stress, and included a postpartum mother and infant follow-up assessment. We hypothesized that COVID-19–related stress would be associated with more maternal mental health problems and higher negativity and lower positivity and orienting responses among infants.

## Methods

The cohort study was approved by the University of New South Wales’ Human Research Ethics Committee. Written informed consent was obtained from each participant or from each parent or guardian. The study is reported in accordance with the Strengthening the Reporting of Observational Studies in Epidemiology (STROBE) reporting guidelines.

### Study Design

The present analysis made use of data from the CORAL study, which includes individuals aged 11 years or older recruited from Australia, the UK, and the US. Participants completed the first survey between May 5 and September 30, 2020, and were invited to complete 2 follow-up surveys at 3 monthly intervals. Individuals who indicated that they were pregnant at time point 1 (T1) were also invited to complete a fourth follow-up survey measuring maternal mental health (ie, symptoms of depression, generalized anxiety disorder [GAD], and postpartum distress) and infant temperament between October 28, 2021, and April 24, 2022 (mean [SD], 17.0 [0.7] months after T1). Participants were recruited via social media, paid advertisings, online pregnancy forums, mothers’ groups newsletters, and mental health organizations. Data were collected via the online survey tool Qualtrics. At T1, every hundredth participant was awarded an A$100 (US$60 or £50) Amazon gift voucher; at the follow-up, all participants were paid an A$20 (US$14 or £12) Amazon gift voucher.

### Participants

A total of 3208 participants completed the CORAL study at T1; 742 participants reported being pregnant at T1, and 342 completed the follow-up survey. Of the 742 participants who were pregnant at T1, 24 subsequently reported a pregnancy loss and were not administered any birth- or infant-related questions at the follow-up. Therefore, the final sample included 318 participants.

### Measures

The following measures were included in the present study. The full list of measures included in the CORAL study at T1 has been reported by Minihan et al.^12^

#### Antenatal COVID-19–Related Stress

The Pandemic Anxiety Scale,^13^ a 9-item questionnaire, was used to measure COVID-19–related stress. The items (eg, “I’m worried that I will catch COVID-19”) were rated on a 5-point Likert scale ranging from 0 (strongly disagree) to 4 (strongly agree), with higher scores indicating greater COVID-19–related stress. The measure demonstrated acceptable internal consistency in the present study (T1: ω total [ω*T*] = 0.86).

#### Symptoms of Depression

Symptoms of depression were assessed with the 8-item Patient Health Questionnaire^14^ (ie, the 9-item Patient Health Questionnaire^14^ excluding the item assessing suicidality because suicide risk could not be managed in the context of an online study). Participants indicated how often they experienced symptoms such as “Little interest or pleasure in doing things” in the previous 2 weeks, measured on a 4-point Likert scale ranging from 0 (not at all) to 3 (nearly every day), with higher scores indicating more frequent symptoms of depression. The measure demonstrated good internal consistency in the present study (T1: ω*T* = 0.91; postpartum: ω*T* = 0.90).

#### Symptoms of GAD

The 7-item General Anxiety Disorder scale (GAD-7)^15^ was administered to assess symptoms of anxiety. Participants indicated how often they experienced symptoms such as “Feeling nervous, anxious, or on edge” over the previous 2 weeks, measured on a 4-point Likert scale ranging from 0 (not at all) to 3 (nearly every day), with higher scores indicating more frequent symptoms of anxiety. The GAD-7 had good internal reliability in the present study (T1: ω*T* = 0.95; postpartum: ω*T* = 0.93).

#### Symptoms of Postpartum Distress

At follow-up, postpartum distress was assessed with the 10-item Postpartum Distress Measure.^16^ The item assessing suicidal ideation was excluded because clinical risk could not be adequately managed online. The scale assessed participants’ agreements with statements such as, “I have recurring thoughts about harm coming to my baby, my family, or myself.” The 9 items were scored on a 4-point Likert scale ranging from 0 (no, this is not true) to 3 (this is true most of the time), with higher scores indicating greater postpartum distress. The scale had good internal consistency in the present sample (ω*T* = 0.90).

#### Infant Temperament

Infant affect and behavior were assessed with the 37-item revised very-short form of the Infant Behavior Questionnaire.^17^ The questionnaire assesses infants’ negative affectivity (eg, “At the end of an exciting day, how often did your baby become tearful?”) and positive affectivity (eg, “When in the bath water, how often did the baby laugh?”) as well as orienting behavior (eg, “How often during the last week did the baby look at pictures in books and/or magazines for 5 minutes or longer at a time?”). In the present study, these items were rated by the mother on a 7-point Likert scale ranging from 1 (never) to 7 (always), with higher scores indicating that the infant exhibited that affect/behavior more frequently. The subscales showed acceptable internal consistency (negative affectivity: ω*T* = 0.90; positive affectivity: ω*T* = 0.78; and orienting behavior: ω*T* = 0.90).

### Statistical Analysis

All analyses were conducted with R, version 4.2.1 (R Group for Statistical Computing)^18^ using the package lme4.^19^ Antenatal COVID-19–related stress^13^ at T1 was included as a fixed effect in models including maternal postpartum mental health and infant temperament outcomes. Linear mixed-effects models were used, in which participant’s scores were nested by country. Missing data were handled using maximum likelihood estimation. Sensitivity analyses were conducted to control for COVID-19 risk experienced during pregnancy (ie, weighted sum indexing COVID-19 diagnoses, hospitalization, and death among respondents and close relations) and infant age at follow-up (range, 8-22 months; mean [SD] age, 13.9 [2.2] months due to variation in gestation at T1). To ensure that COVID-19–related stress did not simply index antenatal maternal mental health, additional sensitivity analyses were conducted controlling for maternal mental health at T1. Statistical tests were 2-sided. A Bonferroni-corrected significance threshold of *P* ≤ .008 was adopted to correct for 6 outcomes. Finally, we explored the interactive effect of infant age and antenatal COVID-19–related stress.

## Results

The results are based on data from 318 women (mean [SD] age, 32.0 [4.6] years) from Australia (88 [28%]), the US (94 [30%]), and the UK (136 [43%]). For a full set of participant characteristics, see Table 1.

**Table 1.** Demographic Information of Participants

Demographic information	No. (%) (N = 318)
Age of mother, mean (SD), y	32.0 (4.6)
Gender identity (% female)	318 (100)
No. of children at T1, mean (SD)	1.2 (0.7)
Country of residence	
Australia	88 (28)
United Kingdom	136 (43)
United States	94 (30)
Race and ethnicity	
Aboriginal or Torres Strait Islander	2 (0.6)
Asian	12 (4)
Hispanic	8 (3)
White	276 (87)
Mixed	7 (2)
Other	10 (3)
Prefer not to say	1 (0.3)
History of psychiatry diagnosis	114 (36)
Highest level of education	
High school	19 (6)
Professional or vocational training	41 (13)
University degree	256 (81)
NA	2 (0.6)
Gestational age at T1, mean (SD) [range], mo	5.8 (2.1) [1-9]
Age of infant at final assessment, mean (SD) [range], mo	13.9 (2.2) [8-22]

Abbreviations: NA, not applicable; T1, time point 1.

In line with previous research,^3^ participants showed depressive and GAD symptoms in the mild (ie, 5-9) clinical range (mean [SD] total score on the 8-item Patient Health Questionnaire for depression, 7.8 [5.4]; mean [SD] total score on the GAD-7 for GAD, 6.7 [5.5]) at T1, and symptoms remained elevated 17 months (ie, 8-22 months post partum) later (mean [SD] score for depression, 7.3 [5.0]; mean [SD] score for GAD, 7.0 [5.3]). Antenatal COVID-19–related stress was significantly associated with depression (β = 0.32 [95% CI, 0.23-0.41]; *P* < .001), GAD (β = 0.35 [95% CI, 0.26-0.44]; *P* < .001), and postpartum distress (β = 0.40 [95% CI, 0.28-0.53]; *P* < .001) among mothers 8 to 22 months post partum (Table 2; Figure). Sensitivity analyses showed that the association of antenatal COVID-19–related stress with maternal postpartum mental health remained significant even after controlling for COVID-19 risk experienced during pregnancy, infant age, and antenatal mental health (eTables 1, 2, and 3 in Supplement 1).

**Table 2.** Association of Antenatal COVID-19–Related Stress With Maternal Mental Health Outcomes

Variable	Anxiety		Depression		Postpartum distress	
	Estimate, β (95% CI)	*P* value	Estimate, β (95% CI)	*P* value	Estimate, β (95% CI)	*P* value
Intercept	0.08 (−1.79 to 1.96)	.93	0.91 (−0.89 to 2.71)	.32	0.29 (−2.35 to 2.92)	.83
Antenatal COVID-19–related stress	0.35 (0.26 to 0.44)	<.001	0.32 (0.23 to 0.41)	<.001	0.40 (0.28 to 0.53)	<.001
Observations	288	NA	288	NA	229	NA
Marginal *R*^2^	0.162	NA	0.151	NA	0.144	NA
Conditional *R*^2^	NA	NA	NA	NA	0.145	NA

Abbreviation: NA, not applicable.

**Figure.**
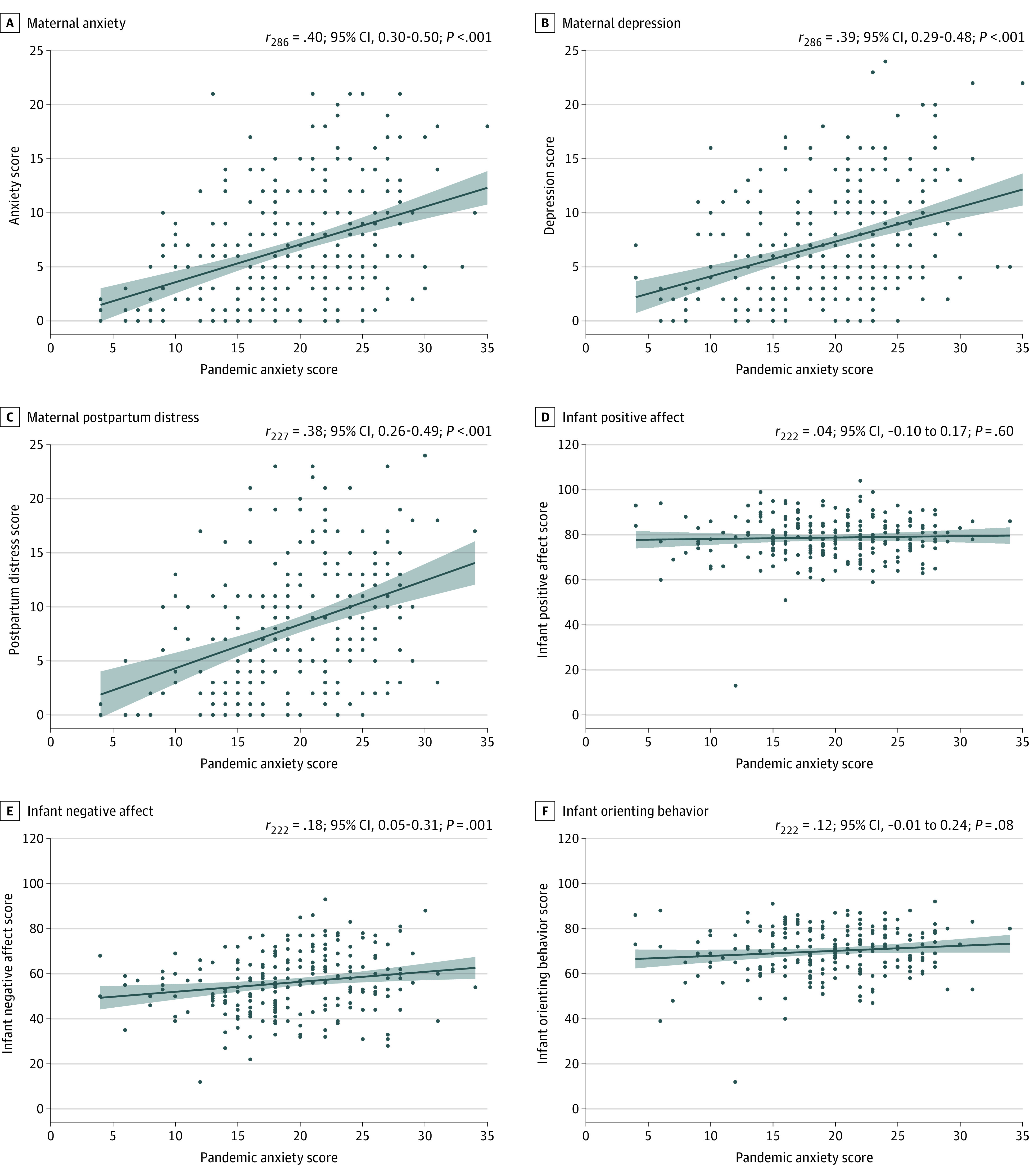
Association Between Antenatal COVID-19–Related Stress With Maternal Mental Health Outcomes and Infant Temperament Outcomes In each case, Pearson correlations are reported above each panel. For full mixed-effects model outputs, see Table 2 and Table 3. The Pandemic Anxiety Scale is a 9-item questionnaire with each item rated 0 to 4, with higher scores indicating greater COVID-19–related stress. Shaded areas indicate 95% CIs.

Among infants, antenatal COVID-19–related stress was selectively associated with negative affectivity (β = 0.45 [95% CI, 0.14-0.76]; *P* = .004) but not positive affect (β = 0.06 [95% CI, −0.17 to 0.29]; *P* = .59) or orienting behaviors (β = 0.22 [95% CI, −0.03 to 0.48]; *P* = .08) (Table 3; Figure; see eTables 4, 5, and 6 in Supplement 1 for sensitivity analyses). The association of antenatal COVID-19–related stress with negative affectivity remained significant when controlling for maternal COVID-19 risk. However, the association of antenatal COVID-19–related stress with negative affectivity no longer reached the Bonferroni-corrected significance threshold of *P* ≤ .008 when controlling for infant age (β = 0.42 [95% CI, 0.11-0.73]; *P* = .009) or maternal symptoms of depression during pregnancy (β = 0.40 [95% CI, 0.06-0.75]; *P* = .02) (eTable 4 in Supplement 1). We further found no interactive association of infant age and antenatal COVID-19–related stress with infant outcomes (eTable 7 in Supplement 1).

**Table 3.** Association of Antenatal COVID-19–Related Stress With Infant Temperament Outcomes

Variable	Negative affectivity		Positive affectivity		Orienting	
	Estimate, β (95% CI)	*P* value	Estimate, β (95% CI)	*P* value	Estimate, β (95% CI)	*P* value
Intercept	47.16 (40.55 to 53.76)	<.001	77.53 (72.86 to 82.21)	<.001	65.62 (60.52 to 70.73)	<.001
Antenatal COVID-19–related stress	0.45 (0.14 to 0.76)	.004	0.06 (−0.17 to 0.29)	.59	0.22 (−0.03 to 0.48)	.08
Observations	224	NA	224	NA	224	NA
Marginal *R*^2^	0.036	NA	0.001	NA	0.014	NA
Conditional *R*^2^	0.055	NA	NA	NA	NA	NA

Abbreviation: NA, not applicable.

## Discussion

The COVID-19 pandemic has exacerbated existing inequalities in mental and physical health outcomes.^20^ This has been especially true for pregnant individuals, who have been disproportionally affected.^4^ Given the long-term adverse effects of poor postpartum mental health on both mother and child, understanding the association of pandemic-related stressors with these outcomes is of paramount importance for public health bodies worldwide. Using data from a longitudinal study across 3 countries, we found a lasting association of antenatal COVID-19–related stress with postpartum maternal mental health and infant negative affectivity.

We also found that COVID-19–related stress was significantly associated with maternal postpartum distress, depression, and GAD as well as infant negative affectivity, even when controlling for COVID-19 risk reported during the pandemic. These results echo the findings of others that antenatal stress is detrimental to the postpartum mental health of both mother and child.^10,11^ More specifically, our findings demonstrate the association of pandemic-related stress with these adverse outcomes, highlighting the need to prioritize mental health care as part of antenatal care guidelines during pandemics.^21^

### Strengths and Limitations

Although our study has a number of strengths—including its longitudinal nature, the inclusion of a validated measure to capture pandemic-related stress, and the inclusion of participants across multiple countries—the findings should be interpreted within the context of its limitations. First, it is possible that preexisting levels of mental health problems among participants may have reduced their ability to cope with environmental stressors, as captured by our measure of COVID-19–related stress. Second, all measures were based on maternal self-report, and we cannot rule out the possibility that mothers who reported heightened COVID-19–related stress during pregnancy expected their child to be struggling more and therefore became overly sensitized to negative cues from their infant. However, this expectancy effect was likely to be minimal in the present study because memory for responses on a 12-item scale from an hour-long survey completed between 8 and 22 months earlier would have been limited. Third, the study was limited by the sample’s lack of diversity, with 276 women (87%) identifying as White and 256 (81%) having a university degree. Future research should include more diverse samples and extend recruitment to pregnant individuals from the Global South.^22^

## Conclusions

The findings of our cohort study make clear the need for further work to examine ways in which we can reduce COVID-19–related stress, or any future pandemic-related stress, to promote mothers’ postpartum mental health and their infants’ well-being. A research agenda needs to be outlined to track the longer-term associations of COVID-19–related stress with maternal and infant outcomes. There is a particular need to identify biological and psychological markers of vulnerability in this population to tailor antenatal care approaches. Pregnant individuals should be classified as a vulnerable group during pandemics as these results show, especially in terms of mental health.

## Notes

Supplement 1. **eTable 1.** The Association of Antenatal COVID-19–Related Stress With Maternal Postpartum Symptoms of Generalized Anxiety **eTable 2.** The Association of Antenatal COVID-19–Related Stress With Maternal Postpartum Symptoms of Depression **eTable 3.** The Association of Antenatal COVID-19–Related Stress With Maternal Postpartum Distress **eTable 4.** The Association of Antenatal COVID-19–Related Stress With Infant Negative Affectivity **eTable 5.** The Association of Antenatal COVID-19–Related Stress with Infant Positive Affectivity **eTable 6.** The Association of Antenatal COVID-19–Related Stress With Infant Orienting Behavior **eTable 7.** The Association Between Antenatal COVID-19–Related Stress With Infant Temperament Outcomes Across Infant Age
eReferences

Supplement 2. **Nonauthor Collaborators.** COVID-19 Risks Across the Lifespan (CORAL) Consortium

Supplement 3.
Data Sharing Statement
