# Supplementary material for: Association of Antenatal COVID-19–Related Stress With Postpartum Maternal Mental Health and Negative Affectivity in Infants
Source: JAMA Netw Open. 2023 Mar 14;6(3):e232969. doi: 10.1001/jamanetworkopen.2023.2969 (PMC10015313; doi:10.1001/jamanetworkopen.2023.2969)
Supplement: Supplement 1. — eTable 1. The Association of Antenatal COVID-19–Related Stress With Maternal Postpartum Symptoms of Generalized Anxiety eTable 2. The Association of Antenatal COVID-19–Related Stress With Maternal Postpartum Symptoms of Depression eTable 3. The Association of Antenatal COVID-19–Related Stress With Maternal Postpartum Distress eTable 4. The Association of Antenatal COVID-19–Related Stress With Infant Negative Affectivity eTable 5. The Association of Antenatal COVID-19–Related Stress with Infant Positive Affectivity eTable 6. The Association of Antenatal COVID-19–Related Stress With Infant Orienting Behavior eTable 7. The Association Between Antenatal COVID-19–Related Stress With Infant Temperament Outcomes Across Infant Age eReferences [file jamanetwopen-e232969-s001.pdf]

Supplementary Online Content

Schweizer S, Andrews JL, Grunewald K, Kumle L; COVID-19 Risks Across the Lifespan (CORAL) Consortium. Association of antenatal COVID-19–related stress with postpartum maternal mental health and negative affectivity in infants. *JAMA Netw Open.* 2023;6(3):e232969. doi:10.1001/jamanetworkopen.2023.2969

eTable 1. The Association of Antenatal COVID-19–Related Stress With Maternal Postpartum Symptoms of Generalized Anxiety
eTable 2. The Association of Antenatal COVID-19–Related Stress With Maternal Postpartum Symptoms of Depression
eTable 3. The Association of Antenatal COVID-19–Related Stress With Maternal Postpartum Distress
eTable 4. The Association of Antenatal COVID-19–Related Stress With Infant Negative Affectivity
eTable 5. The Association of Antenatal COVID-19–Related Stress with Infant Positive Affectivity
eTable 6. The Association of Antenatal COVID-19–Related Stress With Infant Orienting Behavior
eTable 7. The Association Between Antenatal COVID-19–Related Stress With Infant Temperament Outcomes Across Infant Age
eReferences

This supplementary material has been provided by the authors to give readers additional information about their work.

© 2023 Schweizer S et al. *JAMA Network Open.*

eTable 1. The Association of Antenatal COVID-19–Related Stress With Maternal Postpartum Symptoms of Generalized Anxiety

	**Anxiety**			**Anxiety**			**Anxiety**		
Variables	*Estimates*	*CI*	*p*	*Estimates*	*CI*	*p*	*Estimates*	*CI*	*p*
(Intercept)	0.05	-1.83 – 1.94	0.957	0.42	-3.16 – 3.99	0.819	1.16	-0.54 – 2.86	0.180
Antenatal COVID-19-related stress	0.35	0.26 – 0.45	**<0.001**	0.34	0.24 – 0.43	**<0.001**	0.13	0.03 – 0.22	a
COVID-Risk	-0.08	-0.49 – 0.33	0.688						
Infant age				0.00	-0.21 – 0.22	0.985			
Baseline anxiety							0.50	0.40 – 0.60	**<0.001**
**Random Effects**									
σ^2	22.23			22.29			16.44		
τ_{00}	0.00 $_{Country}$			0.00 $_{Country}$			0.00 $_{Country}$		
N	3 $_{Country}$			3 $_{Country}$			3 $_{Country}$		
Observations	287			273			271		
Marginal R^2 / Conditional R^2	0.164 / NA			0.150 / NA			0.379 / NA		

Antenatal COVID-19-related stress was measured using the Pandemic Anxiety Scale (PAS[1]) at T1 (May 2020 to September 2020). This table reports its association with anxiety symptoms on the Generalized Anxiety Disorder-7 (GAD[2]) scale at the postpartum assessment (October 2021 to April 2022). The table reports three models. The first reports a sensitivity analysis including COVID-19 risk experienced during pregnancy at T1. The second reports a sensitivity analysis including infant age. The third model reports a sensitivity analysis including antenatal symptoms of generalized anxiety at T1. A Bonferroni corrected significance level of $p \leq .008$ was used to account for six outcomes across analyses in mothers and infants.

© 2023 Schweizer S et al. *JAMA Network Open.*

eTable 2. The Association of Antenatal COVID-19–Related Stress With Maternal Postpartum Symptoms of Depression

	Depression			Depression			Depression		
Variables	*Estimates*	*CI*	*p*	*Estimates*	*CI*	*p*	*Estimates*	*CI*	*p*
(Intercept)	0.86	-0.95 – 2.66	0.352	0.65	-2.77 – 4.08	0.708	0.66	-0.92 – 2.23	0.413
Antenatal COVID-19-related stress	0.32	0.23 – 0.41	**<0.001**	0.31	0.21 – 0.40	**<0.001**	0.13	0.04 – 0.21	**0.004**
COVID-Risk	0.03	-0.36 – 0.42	0.879						
Infant age				0.04	-0.16 – 0.25	0.670			
Baseline depression							0.52	0.42 – 0.61	**<0.001**
**Random Effects**									
σ^2	20.49			20.45			14.29		
τ_{00}	0.00 $_{Country}$			0.00 $_{Country}$			0.00 $_{Country}$		
N	3 $_{Country}$			3 $_{Country}$			3 $_{Country}$		
Observations	287			273			271		
Marginal R^2 / Conditional R^2	0.152 / NA			0.140 / NA			0.413 / NA		

Antenatal COVID-19-related stress was measured using the Pandemic Anxiety Scale (PAS[1]) at T1 (May 2020 to September 2020). This table reports its association with depressive symptoms on the Patient Health Questionnaire-8 (PHQ-8[3]) scale at the postpartum assessment (October 2021 to April 2022). The table reports three models. The first reports a sensitivity analysis including COVID-19 risk experienced during pregnancy at T1. The second reports a sensitivity analysis including infant age. The third model reports a sensitivity analysis including antenatal symptoms of depression at T1. A Bonferroni corrected significance level of $p \leq .008$ was used to account for six outcomes across analyses in mothers and infants.

© 2023 Schweizer S et al. *JAMA Network Open.*

eTable 3. The Association of Antenatal COVID-19–Related Stress With Maternal Postpartum Distress

	**PP Distress**			**PP Distress**			**PP Distress**		
Variables	*Estimates*	*CI*	*p*	*Estimates*	*CI*	*p*	*Estimates*	*CI*	*p*
(Intercept)	0.27	-2.35 – 2.89	0.841	0.19	-4.88 – 5.25	0.942	0.02	-2.33 – 2.37	0.988
Antenatal COVID-19-related stress	0.40	0.27 – 0.53	**<0.001**	0.40	0.27 – 0.53	**<0.001**	0.20	0.07 – 0.32	**0.002**
COVID-Risk	0.11	-0.40 – 0.63	0.660						
Infant age				0.02	-0.27 – 0.32	0.868			
Baseline depression							0.54	0.41 – 0.67	**<0.001**
**Random Effects**									
σ^2	30.47			30.77			23.04		
τ_{00}	0.02 $_{Country}$			0.00 $_{Country}$			0.00 $_{Country}$		
ICC	0.00								
N	3 $_{Country}$			3 $_{Country}$			3 $_{Country}$		
Observations	229			218			217		
Marginal R^2 / Conditional R^2	0.145 / 0.146			0.142 / NA			0.346 / NA		

Antenatal COVID-19-related stress was measured using the Pandemic Anxiety Scale (PAS[1]) at T1 (May 2020 to September 2020). This table reports its association with maternal postpartum distress measured with the Postpartum Distress Measure[4] at the postpartum assessment (October 2021 to April 2022). The table reports three models. The first reports a sensitivity analysis including COVID-19 risk experienced during pregnancy at T1. The second reports a sensitivity analysis including infant age. The third model reports a sensitivity analysis including antenatal symptoms of depression at T1. A Bonferroni corrected significance level of $p \leq .008$ was used to account for six outcomes across analyses in mothers and infants.

© 2023 Schweizer S et al. *JAMA Network Open.*

eTable 4. The Association of Antenatal COVID-19–Related Stress With Infant Negative Affectivity

	Negative Affectivity			Negative Affectivity			Negative Affectivity		
Variables	*Estimates*	*CI*	*p*	*Estimates*	*CI*	*p*	*Estimates*	*CI*	*p*
(Intercept)	46.81	40.14 – 53.48	**<0.001**	58.51	46.46 – 70.56	**<0.001**	47.15	40.20 – 54.11	**<0.001**
Antenatal COVID-19-related stress	0.43	0.12 – 0.74	**0.006**	0.42	0.11 – 0.73	0.009	0.40	0.06 – 0.75	0.023
COVID-Risk	1.37	0.08 – 2.66	0.037						
Infant age				-0.72	-1.41 – -0.04	0.039			
Baseline depression							0.14	-0.22 – 0.50	0.454
**Random Effects**									
σ^2	167.09			167.66			173.20		
τ_{00}	4.69 Country			2.37 Country			4.69 Country		
ICC	0.03			0.01			0.03		
N	3 Country			3 Country			3 Country		
Observations	224			214			213		
Marginal R^2 / Conditional R^2	0.055 / 0.081			0.051 / 0.064			0.038 / 0.063		

Antenatal maternal COVID-19-related stress was measured using the Pandemic Anxiety Scale (PAS[1]) at T1 (May 2020 to September 2020). This table reports its association with infant negative affectivity measured with the Infant Behavior Questionnaire[5] at the postpartum assessment (October 2021 to April 2022). The table reports three models. The first reports a sensitivity analysis including COVID-19 risk experienced during pregnancy at T1. The second reports a sensitivity analysis including infant age. The third model reports a sensitivity analysis including antenatal, maternal symptoms of depression at T1. A Bonferroni corrected significance level of $p \leq .008$ was used to account for six outcomes across analyses in mothers and infants.

© 2023 Schweizer S et al. *JAMA Network Open.*

eTable 5. The Association of Antenatal COVID-19–Related Stress with Infant Positive Affectivity

	Positive Affectivity			Positive Affectivity			Positive Affectivity		
Variables	*Estimates*	*CI*	*p*	*Estimates*	*CI*	*p*	*Estimates*	*CI*	*p*
(Intercept)	77.57	72.90 – 82.25	**<0.001**	80.38	71.35 – 89.40	**<0.001**	77.05	72.24 – 81.87	**<0.001**
Antenatal COVID-19-related stress	0.07	-0.16 – 0.30	0.559	0.08	-0.16 – 0.31	0.509	0.13	-0.13 – 0.39	0.321
COVID-Risk	-0.25	-1.21 – 0.71	0.610						
Infant age				-0.22	-0.74 – 0.30	0.400			
Baseline depression							-0.11	-0.37 – 0.16	0.437
**Random Effects**									
σ^2	95.72			96.20			95.81		
τ_{00}	0.00 $_{Country}$			0.00 $_{Country}$			0.00 $_{Country}$		
N	3 $_{Country}$			3 $_{Country}$			3 $_{Country}$		
Observations	224			214			213		
Marginal R^2 / Conditional R^2	0.002 / NA			0.005 / NA			0.005 / NA		

Antenatal maternal COVID-19-related stress was measured using the Pandemic Anxiety Scale (PAS[1]) at T1 (May 2020 to September 2020). This table reports its association with infant positive affectivity measured with the Infant Behavior Questionnaire[5] at the postpartum assessment (October 2021 to April 2022). The table reports three models. The first reports a sensitivity analysis including COVID-19 risk experienced during pregnancy at T1. The second reports a sensitivity analysis including infant age. The third model reports a sensitivity analysis including antenatal, maternal symptoms of depression at T1. A Bonferroni corrected significance level of $p \leq .008$ was used to account for six outcomes across analyses in mothers and infants.

© 2023 Schweizer S et al. *JAMA Network Open.*

eTable 6. The Association of Antenatal COVID-19–Related Stress With Infant Orienting Behavior

	**Orienting**			**Orienting**			**Orienting**		
Variables	*Estimates*	*CI*	*p*	*Estimates*	*CI*	*p*	*Estimates*	*CI*	*p*
(Intercept)	65.65	60.54 – 70.76	**<0.001**	68.98	59.25 – 78.71	**<0.001**	65.54	60.34 – 70.74	**<0.001**
Antenatal COVID-19-related stress	0.23	-0.02 – 0.48	0.076	0.22	-0.04 – 0.47	0.093	0.37	0.10 – 0.65	**0.008**
COVID-Risk	-0.18	-1.23 – 0.88	0.739						
Infant age				-0.23	-0.79 – 0.33	0.417			
Baseline depression							-0.34	-0.63 – -0.05	0.020
**Random Effects**									
σ^2	114.48			111.04			111.45		
τ_{00}	0.00 Country			0.45 Country			0.00 Country		
ICC				0.00					
N	3 Country			3 Country			3 Country		
Observations	224			214			213		
Marginal R^2 / Conditional R^2	0.014 / NA			0.016 / 0.020			0.041 / NA		

Antenatal maternal COVID-19-related stress was measured using the Pandemic Anxiety Scale (PAS[1]) at T1 (May 2020 to September 2020). This table reports its association with infant orienting behavior measured with the Infant Behavior Questionnaire[5] at the postpartum assessment (October 2021 to April 2022). The table reports three models. The first reports a sensitivity analysis including COVID-19 risk experienced during pregnancy at T1. The second reports a sensitivity analysis including infant age. The third model reports a sensitivity analysis including antenatal, maternal symptoms of depression at T1. A Bonferroni corrected significance level of $p \leq .008$ was used to account for six outcomes across analyses in mothers and infants.

© 2023 Schweizer S et al. *JAMA Network Open.*

eTable 7. The Association Between Antenatal COVID-19–Related Stress With Infant Temperament Outcomes Across Infant Age

	Negative Affectivity			Positive Affectivity			Orienting		
Variables	*Estimates*	*CI*	*p*	*Estimates*	*CI*	*p*	*Estimates*	*CI*	*p*
(Intercept)	71.50	34.47 – 108.53	**<0.001**	100.71	72.88 – 128.55	**<0.001**	97.49	67.64 – 127.34	**<0.001**
Antenatal COVID-19-related stress	-0.26	-2.10 – 1.59	0.786	-0.98	-2.37 – 0.41	0.167	-1.26	-2.75 – 0.23	0.097
Infant age	-1.60	-4.06 – 0.86	0.201	-1.59	-3.44 – 0.26	0.091	-2.15	-4.14 – -0.17	0.034
Antenatal COVID-19-related stress X Infant age	0.05	-0.08 – 0.17	0.466	0.07	-0.02 – 0.16	0.130	0.10	0.00 – 0.20	0.048
**Random Effects**									
σ^2	167.37			95.17			109.39		
τ_{00}	2.13 $_{Country}$			0.00 $_{Country}$			0.06 $_{Country}$		
ICC	0.01						0.00		
N	3 $_{Country}$			3 $_{Country}$			3 $_{Country}$		
Observations	214			214			214		
Marginal R^2 / Conditional R^2	0.053 / 0.065			0.016 / NA			0.034 / 0.035		

Antenatal COVID-19-related stress was measured using the Pandemic Anxiety Scale (PAS) at T1 (May 2020-September 2020). This table reports its association with infant negative and positive affectivity and orienting behavior at the postpartum assessment (October 2021 - April 2022). And whether the effect of Antenatal COVID-19-related stress varies as a function of infant age. A Bonferroni corrected significance level of $p \leq .008$ was used to account for six outcomes across analyses in mothers and infants.

© 2023 Schweizer S et al. *JAMA Network Open.*

eReferences

1. McElroy E, Patalay P, Moltrecht B, et al. Demographic and health factors associated with pandemic anxiety in the context of COVID-19. *Br J Health Psychol*. 2020;25(4):934-944. doi:10.1111/bjhp.12470

2. Spitzer RL, Kroenke K, Williams JBW, Löwe B. A Brief Measure for Assessing Generalized Anxiety Disorder: The GAD-7. *Arch Intern Med*. 2006;166(10):1092. doi:10.1001/archinte.166.10.1092

3. Kroenke K, Spitzer RL, Williams JBW. The PHQ-9. *J Gen Intern Med*. 2001;16(9):606-613. doi:10.1046/j.1525-1497.2001.016009606.x

4. Allison KC, Wenzel A, Kleiman K, Sarwer DB. Development of a brief measure of postpartum distress. *J Womens Health*. 2011;20(4):617-623. doi:10.1089/jwh.2010.1989

5. Putnam SP, Helbig AL, Gartstein MA, Rothbart MK, Leerkes E. Development and Assessment of Short and Very Short Forms of the Infant Behavior Questionnaire–Revised. *J Pers Assess*. 2014;96(4):445-458. doi:10.1080/00223891.2013.841171

© 2023 Schweizer S et al. *JAMA Network Open*.
