# Supplementary material for: Association of Antenatal COVID-19–Related Stress With Postpartum Maternal Mental Health and Negative Affectivity in Infants
Source: JAMA Netw Open. 2023 Mar 14;6(3):e232969. doi: 10.1001/jamanetworkopen.2023.2969 (PMC10015313; doi:10.1001/jamanetworkopen.2023.2969)
Supplement: Supplement 2. — Nonauthor Collaborators. COVID-19 Risks Across the Lifespan (CORAL) Consortium [file jamanetwopen-e232969-s002.pdf]

Supplemental Online Content: Nonauthor Collaborators
*First name, last name, and suffix (if applicable) are required and will appear in PubMed.

*Group Name(s): COVID-19 Risks Across the Lifespan (CORAL) Consortium							
***First Name and Middle Initial(s)**	***Last Name**	***Suffix (eg, Jr, III)**	Academic Degrees	Institution	Location (city, state/province, country)	Role or Contribution, eg, chair, principal investigator	Group (if more than 1 Group listed in the byline) and/or Subgroup (eg, Steering Committee)
Savannah	Minihan			University of New South Wales	Sydney, Australia		
Annabel	Songco			University of New South Wales	Sydney, Australia		
Elaine	Fox			University of Adelaide	Adelaide, Australia		
Cecile D.	Ladouceur			University of Pittsburgh	Pittsburgh, Pennsylvania		
Louise	Mewton			University of New South Wales	Sydney, Australia		
Michelle	Moulds			University of New South Wales	Sydney, Australia		
Jennifer H.	Pfeifer			University of Oregon	Eugene, Oregon		
Anne-Laura	van Harmelen			Leiden University Leiden	Netherlands		
