# Supplementary material for: Association of Antenatal COVID-19–Related Stress With Postpartum Maternal Mental Health and Negative Affectivity in Infants
Source: JAMA Netw Open. 2023 Mar 14;6(3):e232969. doi: 10.1001/jamanetworkopen.2023.2969 (PMC10015313; doi:10.1001/jamanetworkopen.2023.2969)
Supplement: Supplement 3. — Data Sharing Statement [file jamanetwopen-e232969-s003.pdf]

Data Sharing Statement

Schweizer. Association of Antenatal COVID-19-Related Stress With Postpartum Maternal Mental Health and Negative Affectivity in Infants. *JAMA Netw Open*. Published March 14, 2023. doi:10.1001/jamanetworkopen.2023.2969

Data
Data available: Yes
Data types: Deidentified participant data
How to access data: Data will be made available upon reasonable request to the first author: s.schweizer@unsw.edu.au.
When available: With publication

Supporting Documents
Document types: None

Additional Information
Who can access the data: Resarchers whose proposed use of the data has been approved
Types of analyses: Specified purpose as per the approval
Mechanisms of data availability: After approval of proposal
